# A Molecular Epidemiology Survey of Respiratory Adenoviruses Circulating in Children Residing in Southern Palestine

**DOI:** 10.1371/journal.pone.0042732

**Published:** 2012-08-03

**Authors:** Lina Qurei, Donald Seto, Zaidoun Salah, Maysa Azzeh

**Affiliations:** 1 Virology Research Laboratory, Medical Research Center, Al-Quds University , East Jerusalem-Abu Dies, Palestine; 2 School of Systems Biology, George Mason University, Manassas, Virginia, United States of America; French National Centre for Scientific Research, France

## Abstract

A molecular epidemiology survey was performed in order to establish and document the respiratory adenovirus pathogen profiles among children in Southern Palestine. Three hundred and thirty-eight hospitalized pediatric cases with adenovirus-associated respiratory tract infections were analyzed. Forty four cases out of the 338 were evaluated in more detail for the adenoviruses types present. All of the children resided in Southern Palestine, that is, in city, village and refugee camp environments within the districts of Hebron and Bethlehem. Human adenoviruses circulated throughout 2005–2010, with major outbreaks occurring in the spring months. A larger percent of the children diagnosed with adenoviral infections were male infants. DNA sequence analysis of the hexon genes from 44 samples revealed that several distinct adenovirus types circulated in the region; these were HAdV-C1, HAdV-C2, HAdV-B3 and HAdV-C5. However, not all of these types were detected within each year. This is the first study ever conducted in Palestine of the genetic epidemiology of respiratory adenovirus infections.

## Introduction

Human adenoviruses (HAdVs) are classified within the family Adenoviridae, genus Mastadenovirus. Based on their hemagglutination and serum neutralization properties, 51 serotypes of HAdVs were orginally identified [1]. However, a more recent and comprehensive understanding of HAdVs has been made possible by the use of genomics and bioinformatics. These approaches are used to identify and characterize novel human adenoviruses based on the whole genome sequence, extending beyond the hexon and fiber markers, and phylogenomics [2]. As a result, additional seven types have been identified, including several novel respiratory and ocular pathogens. These new HAdV genotypes were identified as either sequence-divergent and/or recombinant viruses of previously recognized types, and include HAdV-G52 [3]; HAdV-D53 [4]; HAdV-D54 [5]; HAdV-B55 [6]; HAdV-D56 [7]; HAdV-D57 [8]; and HAdV-D58 [9].

HAdVs were initially characterized as respiratory tract pathogens in the 1950s [10], [11]. Although respiratory infections caused by HAdVs are mostly self-limiting, severe and even fatal infections may occur. HAdV infections in children predominantly involve the respiratory tract, with approximately 5–7% of respiratory illnesses in young children attributed to HAdVs [12], [13]. Particular HAdV species and serotypes are correlated with specific diseases, epidemiologic settings and demographic risk groups [14]. Infections of the respiratory tract are predominantly caused by HAdV types of species B, C and E; however, severe acute respiratory disease is mainly caused by only few types, namely HAdV-B3, -E4, -B7, -B14, -B16, -B21 and -B55 [6], [14], [15], [16], [17]. In addition, HAdVs are also responsible for a wide spectrum of clinical diseases involving ocular, gastrointestinal and genitourinary tissues [14].

Presented in this report are data from the epidemiological analysis of 338 cases of HAdV isolated from nasal pharyngeal aspirates (NPAs). These were sampled from hospitalized pediatric patients who resided in Southern Palestine between 2005 and 2010. Of these, 44 samples were analyzed in detail using DNA sequence-based HAdV typing to illuminate the viruses circulating in Southern Palestine. For this, a nested PCR and DNA sequencing assay of the HAdV hexon gene HVR1–6, developed by Lu and Erdman [18] and successfully used by several researchers [19], [20], [21], [22], was utilized. Our work reveals which HAdV types were circulating in southern Palestine during the study period, allowing for strategies in developing and applying potential vaccines.

## Methods

### Ethics Statement

Patients' data collection and archived patients' samples were approved specifically for this research by the Human Medical Research Committee of the Caritas Baby Hospital in a written approval letter dated March, 19th, 2010.

### Study population

**Figure 1:**
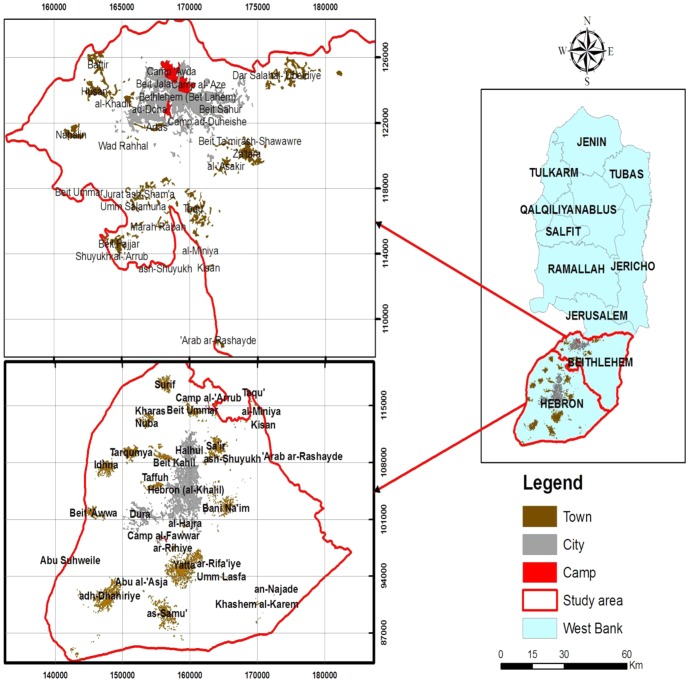
A map of the West Bank and a detailed map of Southern Palestine. The detailed maps of Southern Palestine on the left show the villages and refugee camps in the districts of Hebron and Bethlehem, where the children with respiratory tract infection caused by Adenovirus reside.

The respiratory pathogens were sampled from patients admitted to the Caritas Baby Hospital (CBH), which is located in Bethlehem. Accordingly, most of the children admitted came from the southern regions of Palestine, particularly the districts of Bethlehem and Hebron. These children represent a cross-section of diverse living conditions: cities, refugee camps and villages throughout the region. Figure 1 indicates the areas in which the children reside, for reference. Between January 2005 and December 2010, 8033 respiratory tract infection cases residing in southern Palestine were admitted to the CBH, 338 cases (4.2%) were caused by HAdV. The other cases were caused by other respiratory tract pathogens. The ages of the children ranged from a few days to seven years.

### Demographics and medical records of patients

Data from all of the 338 pediatric patients in this study were subjected to statistical analysis, including the parameters of age, sex, residency and date of admission, as collected from the CBH records. Additionally, clinical data, including symptoms and other metadata, were collected from the medical charts of 150 children. All data were entered into a Microsoft Excel spreadsheet for statistical analysis. The statistical associations of the data were concluded using Pearson's Chi-squared test for independence. The analyses were performed using statistical package for social sciences (SPSS) software.

### Nasopharyngeal aspirate specimen

The nasopharyngeal aspirates (NPAs) were collected at the CBH from the children between 2005 and 2010 and archived. These were tested for respiratory tract viruses, using a direct Immunofluorescence assay. This test was performed at the CBH clinical laboratory using Millipore Respiratory DFA Viral Screening & Identification Kit (Light Diagnostics; CA, USA). This kit identifies HAdV, Respiratory Synsytial Virus (RSV), Parainfluenza (1, 2 and 3) and Influenza (A and B). In this study, one hundred archived NPA samples, tested positive for HAdV, were subjected to the molecular genotyping of HAdV by DNA sequencing. These 100 NPA samples were selected randomly and represented 10–20 NPAs per year (2005–2010).

### DNA extraction and PCR

DNA extraction from 100 NPA was performed using the QiaAmp DNA mini kit (Qiagen 51304), according to the manufacturer's instructions. AdhexF1/R1 and AdhexF2/R2 primer pairs [18] were used in one PCR reaction to amplify a ∼750 bp of the hexon gene as followed: 94°C for 2 min, 35 cycles of 94°C for 1 min, 45°C for 1 min, and 72°C for 2 min with a final extension of 72°C for 5 min. PCR products were purified using the QiaAmp PCR Purification Kit according to the manufacturer's instructions (Qiagen 28004), and then subjected to DNA sequencing using Sanger chemistry (ABI 3130; Applied Biosystems, Inc., USA).

### Sequence analysis and HAdV typing

Forty-four DNA sequences, representing 13% of the total HAdV cases which were distributed throughout the study years, were analyzed using the NCBI BLAST software (http://www.ncbi.nlm.nih.gov/blast/Blast.cgi?PAGE=Nucleotides) and the DNASTAR Lasergene MegAlign program (DNASTAR Inc., WI, USA) using reference sequences.

## Results

### Residency, sex and age distribution of HAdV-infected children

**Figure 2:**
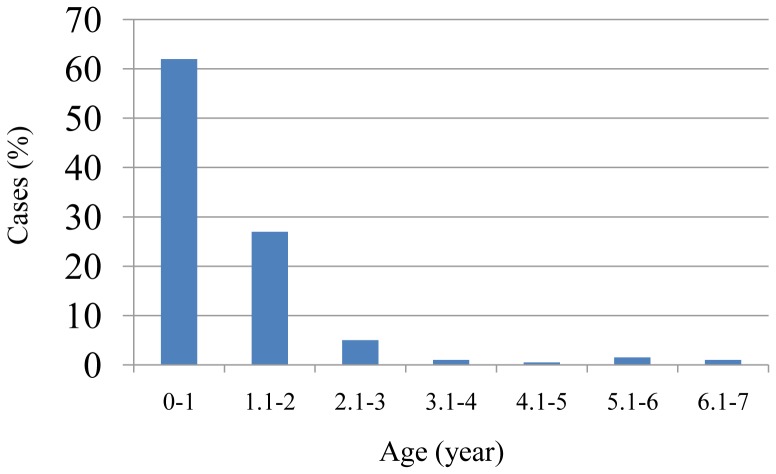
The percentage of pediatric cases suffering from HADV for age groups between a few days and 7 years. The percentage was calculated according to the 338 total number of HAdV cases investigated in this study.

The pediatric patients were predominantly from villages (68%), while 21% were residents of refugee camps and 11% were from cities. A larger percentage of children diagnosed with HAdV respiratory tract infections were male (60%). The children, with ages ranging between a few days and one year (age group 0–1), showed the highest rate of HAdV infections (62%), followed by the age group 1.1–2 years (Fig. 2).

### Yearly and seasonal HAdV infection

**Figure 3:**
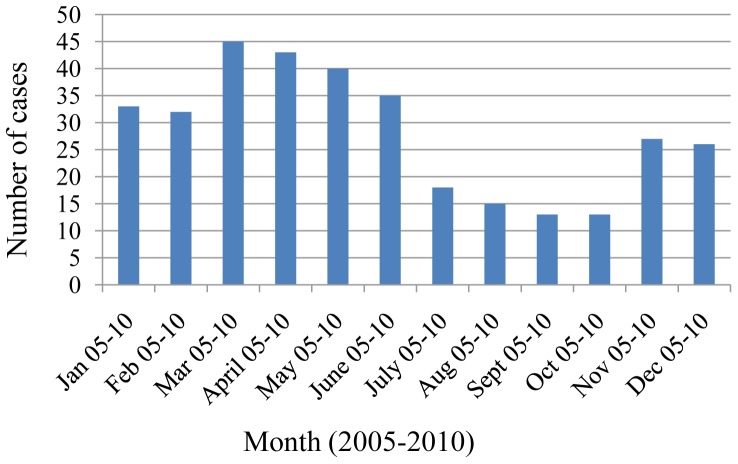
HAdV cases per month from 2005–2010. The actual number of cases each month and for each year is displayed along the y-axis.

HAdV infections increased yearly, with 40 cases detected in 2005 and almost 100 cases noted in 2010. These infections occurred throughout the year, with a detectable increase of cases in the months ranging from March to May (Fig. 3). In 2010, the year with the highest recorded number of HAdV infections, however, most cases occurred in January. Pearson's Chi-squared test was used to test for an association between HAdV infection and season, the test was significant (P value<0.001).

### Clinical observations

Metadata from the medical charts revealed 150 cases belonging to the group of children with the highest HAdV infection rate, that is, aged one year and younger. An average of 25 cases per year were distributed over the four seasons, with the majority of these cases sexed male (71%). A majority of the children (80%) suffered from fevers as a major sign of lower respiratory disease; whereas only 50% suffered from coughs. Thirty-three percent had severe symptoms, requiring mechanical ventilation. Bronchitis/bronchopneumonia (33.3%) and upper respiratory tract infections (URTI) (24.7%) were the major presenting symptoms in the children upon admission. The following symptoms were also observed, with a lower frequency of 2–5%: sepsis, gastroenteritis, otitis media, meningitis, dehydration, tonsillitis, congenital heart disease (CHD), conjunctivitis, chronic renal failure, yellow skin and febril convulsion. In individual cases, the children presented with croup, skin rash, hypoglycemia, anemia, cystic leukomalacia and lung infection. None of the 150 charts recorded co-infection with another respiratory tract virus. Finally, 104 cases (69.3%) were treated with antibiotics as documented in the medical records. Of these, 70 children were indeed suffering from bacterial co-infections; therefore antibiotics should have not been prescribed in 34 cases (33% of those children who actually received antibiotics) as they had exclusively viral infection.

### Genetic analysis of HAdV circulating in Southern Palestine

**Figure 4:**
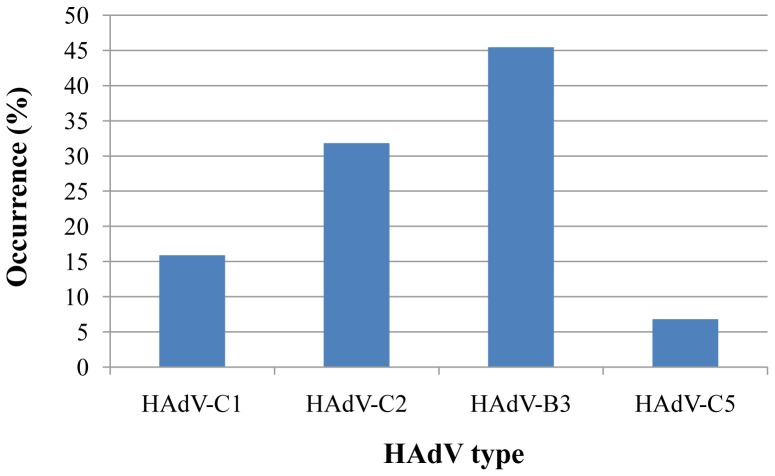
Circulating HAdV types in southern Palestine. HAdV types circulating in Southern Palestine from 2005 to 2010 were detected using nested PCR and DNA sequencing assay of the HAdV hexon gene HVR1–6 followed by sequencing and sequence analysis. The 44 sequences were assigned the GenBank accession numbers [JQ796022–JQ796065]. The percentage of each serotype was calculated relatively to the number of total sequenced samples (44 samples).

The most common HAdV types as revealed from comparing Palestinian sequences with reference data were HAdV-B3 and HAdV-C2, represented in 45.5% and 31.8% of the 44 sequenced samples respectively (Fig. 4). Less dominant were other types of C: HAdV-C1 and HAdV-C5, which were represented in 15.9% and 6.8% of the samples, respectively (Fig. 4). Overall, species C was the most common HAdV species among hospitalized children in Southern Palestine (54.5%).

### Annual distribution of human adenovirus serotypes

The different types of HAdV were not present equally across the years. While HAdV-C2 was present throughout the years of the study, HAdV-C1 was present in all years except 2008; HAdV-B3 was present in all years except 2009, and HAdV-C5 was present only in 2005 and 2006 (Table 1). HAdV-B3 was the most dominant type in 2005 and 2010, as it presented in 62% and 71% of the samples respectively. HAdV-C2 was dominant in 2009, occurring in 67% of the samples. In the other years, 2006–2008, no specific HAdV type seemed to dominate.

**Table 1:** Yearly presence of HAdV types in southern Palestine.

Serotypes	2005	2006	2007	2008	2009	2010
HAdV-C1	+	+	+	_	+	+
HAdV-C2	+	+	+	+	+	+
HAdV-B3	+	+	+	+	_	+
HAdV-C5	+	+	_	_	_	_

Viruses isolated from pediatrics patients in a six-year period were subjected to DNA sequencing of the hexon gene, with BLAST analysis using the NCBI software (http://www.ncbi.nlm.nih.gov/blast/Blast.cgi?PAGE=Nucleotides) and the DNASTAR program using reference sequences. Not all types are represented each year, with HAdV-C5 absent from 2007 onward.

## Discussion

Adenovirus is a significant causative agent of respiratory tract disease in pediatric and adult patients. Numerous outbreaks of acute respiratory disease caused by HAdV have been reported during the last decade in many countries [17], [23], [24], [25], [26], [27], [28], [29], [30], [31], [32], [33]. Some outbreaks resulted in high morbidity and mortality in neonatal and pediatric units, in children admitted to hospitals [32], [34], [35], [36], [37], [38], in schools [17] and most strikingly in military camps [26], [39], [40] and military hospitals [41]. Presented in this report are epidemiological and limited DNA sequence analyses of HAdV respiratory tract infections from hospitalized children, who resided in Southern Palestine between 2005 and 2010. The HAdV infection rate among other respiratory tract infections in this study population was 4.2%, in line with earlier reports [12], [13]. The highest percentage (68%) of infections among children in this sample population with adenovirus was located in villages. This may be due to population density and the living conditions coupled with the high potential for contact between children in the village community, along with their lower socioeconomic status. Adults and children of lower socioeconomic status are known to be at higher risk for a wide range of contagious infectious diseases, especially respiratory [42]. Surprisingly, the number of cases from refugee camps was the lowest in this study, which may be partially explained by the camp residents taking advantages of clinical services provided by the UN. Accordingly, Pearson's Chi-squared test for independence used to measure the association between adenovirus infection and district residency was not significant (P value>0.05). The higher rate of male infections of HAdV found in this study was previously described [43], [44]. Interestingly, the lowest HAdV infection rate was among school age children (age group 6.1–7 years). This may suggest that they had already been exposed to the common endemic serotypes of adenovirus early in life and may have thereby established a protective immunity [43]. Children younger than two years of age were the most susceptible to respiratory tract infections of HAdV (89% of total cases). According to the Pearson's Chi-squared test for independence, a strong association between age and HAdV infection was present in our study (P value<0.001). Adenovirus outbreaks were more likely to occur in young children and infants, as confirmed by earlier reports [32], [34], [35], [36], [37], [38].

The HAdV-associated respiratory diseases, as documented in medical charts, were bronchitis/bronchopneumonia (33.3%) as well as upper respiratory tract infections URTI (24.7%). Indeed, the most common clinical presentations of HAdV infections are pneumonia, bronchitis/bronchiolitis, conjunctivitis, pharyngoconjunctival fever and upper respiratory tract infections or common cold-like symptoms [25], [26], [34], [45], [46], [47], [48], [49], [50], [51]. A significant association of symptoms was found between fevers and HAdV infection (P value<0.001), as well as cough and HAdV infections (P value<0.001). Coughs and fevers are common symptoms related to various respiratory tract infections [26] and respiratory tract infections of HAdV [43], [49], [52]. As antibiotics may be useful in some cases, in order to prevent further complications caused by bacterial co-infections due to weakened immunity during viral infections or in case of viral co-infection with bacterial agents [53], [54], 34 children received these prophylactic antibiotics, despite the exclusive viral URTI assayed. In general, prescribing antibiotics before diagnosis or because an exact diagnosis is not possible is a common mistake made by most physicians, and is a practice that has led to more and more drug-resistant bacterial strains worldwide along with unexpected complications in patients [54], [55], [56], [57]. Therefore, admittedly, the diagnostics of viral respiratory infections in medical laboratories in Palestine is substandard and is a major problem that could have global impact in this era of a “global village”.

In conclusion, this study demonstrated that HAdV-C1, HAdV-C2, HAdV-B3 and HAdV-C5 were the most common HAdV types circulating among hospitalized children in Southern Palestine currently and recently. The different types were not equally distributed over all the years or all of the months in the year sampled. It has been shown earlier that some HAdV types correlated specifically to one or two months throughout the year, or were also found in some years but not in others [43]. Similar genetic epidemiological studies have shown that HAdV types, comprising HAdV-C1, -C2, -B3, -C5, -C6, and -B7 were mainly involved in respiratory tract infections in children living in Argentina, Iceland, Chile, Taiwan, Canada, China, Malaysia, Korea, Israel, Egypt and the USA, respectively [17], [22], [25], [31], [51], [52], [58], [59], [60], [61], [62]. HAdV types -C1, -C2 and -B3 were detected in most of these studies. HAdV-C5, however, was detected in Iceland, Taiwan, Korea, Egypt and according to a very recent study, also in Argentina [31], [52], [59], [62], [63]. Interestingly, the prevalence of HAdV-C5 in all of these studies was low, in line with our findings. With regards to the noted HAdV type predominance of HAdV-B3, followed by HAdV-C1, -C2 and -C5, our current study is consistent with a regional study reported in Egypt [62]. The most striking co-accordance between our study and the Egyptian one was the fact that HAdV-C5 was only detected in two out of four years of their study period [62].

Although most studies of HAdV infections were unable to show a clear evidence for seasonal distribution, specific types have been found to correlate to specific seasons [43], [64]. Also presented in this report, HAdV-C2 and HAdV-B3 seem to occur more frequently in the spring and winter seasons. Nevertheless, this finding needs further confirmation by the analysis of a larger number of HAdV samples throughout the study years 2005–2010. Of all the HAdV types detected in Southern Palestine, only HAdV-B3 has been identified as well in numerous other studies to be responsible for respiratory disease outbreaks; for example, in the USA, Taiwan, Korea and Portugal [24], [28], [31], [32]. Although there were no records of an equivalent outbreak in Southern Palestine, the adenovirus typing results show clearly that HAdV-B3 was the most common serotype over all the years with 45.5%, and presenting in 68% of the samples in the years 2005 and 2010. These data may provide evidence for a possible unrecorded and undocumented outbreak, since HAdV-B3 was also reported to be detected in both outbreaks and in sporadic cases [31]. In summary, this is the first study ever to provide an insight into the epidemiological characteristics and genotype profile of HAdV types in Palestine, including a breakdown of cities, refugee camps and villages throughout Southern Palestine. Detection of the circulating HAdV types contributing to respiratory tract infections in Palestine allows for future vaccination strategies in the region. Finally, the use of DNA sequencing-based techniques for typing viral infection agents and its application in Palestine presents a solid platform for investigating viral infections and will be very valuable in the case of respiratory outbreaks.
